# Rab32 Is Important for Autophagy and Lipid Storage in *Drosophila*

**DOI:** 10.1371/journal.pone.0032086

**Published:** 2012-02-14

**Authors:** Chao Wang, Zhonghua Liu, Xun Huang

**Affiliations:** 1 State Key Laboratory of Molecular Developmental Biology, Institute of Genetics and Developmental Biology, Chinese Academy of Sciences, Beijing, China; 2 Graduate School of the Chinese Academy of Sciences, Beijing, China; Clermont Université, France

## Abstract

Lipids are essential components of all organisms. Within cells, lipids are mainly stored in a specific type of organelle, called the lipid droplet. The molecular mechanisms governing the dynamics of lipid droplets have been little explored. The protein composition of lipid droplets has been analyzed in numerous proteomic studies, and a large number of lipid droplet-associated proteins have been identified, including Rab small GTPases. Rab proteins are known to participate in many intracellular membranous events; however, their exact role in lipid droplets is largely unexplored. Here we systematically investigate the roles of *Drosophila* Rab family proteins in lipid storage in the larval adipose tissue, fat body. Rab32 and several other Rabs were found to affect the size of lipid droplets as well as lipid levels. Further studies showed that Rab32 and Rab32 GEF/Claret may be involved in autophagy, consequently affecting lipid storage. Loss-of-function mutants of several components in the autophagy pathway result in similar effects on lipid storage. These results highlight the potential functions of Rabs in regulating lipid metabolism.

## Introduction

Lipids, proteins and carbohydrates are the three major building components of all living organisms. Lipids provide energy for daily usage and also function as signaling molecules in the regulation of important biological processes [1]. To maintain proper physiological conditions, the metabolism and homeostasis of lipids must be precisely regulated. Defects in lipid metabolism can lead to health-threatening problems in humans, for example, obesity and insulin resistance [2], [3], [4].

In most animals, storage lipids are usually accumulated in adipose tissues. Within cells, neutral lipids, mainly triacylglycerol (TAG) and cholesterol ester (CE), are stored in a specific type of organelle, called the lipid droplet [5]. Under nutrient-rich situations, excess fatty acids can be converted to TAG through lipogenesis and stored in lipid droplets. Under some nutrient-limiting conditions such as starvation, lipids can be released from lipid droplets by lipolysis for cell usage. Maintaining the homeostasis of lipid droplets is therefore important for normal lipid metabolism and lipid-related diseases.

Lipid droplets contain a lipid core and a monolayer of protein-coated phospholipid membrane [6]. The size and the content of lipid droplets are largely regulated by the balance of lipogenesis and lipolysis, which is mediated by many lipases. PAT (Perilipin/APRP/TIP47) domain proteins, the best known lipid droplet-surface proteins, can interact with lipases [7]. PAT proteins regulate the lipid droplet surface access of lipase to modulate the lipolysis process [8], [9]. Many fundamental aspects of the dynamics of lipid droplets, including their biogenesis, the transport of lipids in and out of lipid droplets, and intracellular trafficking of lipid droplets, are not well characterized.

Identifying the proteins involved in these processes will lead to a better understanding of the dynamics of lipid droplets. Lipid droplets from different types of cells/tissues in several organisms have been purified and many proteomic studies have been conducted to identify proteins associated with them [10], [11], [12]. These proteins are likely localized on the surface of lipid droplets and function directly in lipid droplet dynamics. Many members of the Rab small GTPase family have been associated with lipid droplets in proteomic studies [13], [14]. Rab family proteins are known to function in intracellular membrane trafficking. They participate in many biological processes, including endocytosis and exocytosis, cytokinesis, melanosome formation, autophagosome formation, lysosome biogenesis, and signaling transduction [15]. The identification of Rabs in lipid droplet proteomic studies suggests a potential role for Rabs in regulating the dynamics of lipid droplets and/or lipid storage. Indeed, the recruitment of Rab18 to lipid droplets is regulated by the metabolic state of lipid droplets, implying that Rab18 may mobilize lipids stored in lipid droplets [16]. Although a start has been made in exploring the roles of Rab18 in lipid droplets, the functions of the majority of Rab proteins identified in proteomic studies in lipid storage are still unclear. In addition, it is possible that other Rabs which have not yet been identified in proteomic studies may also have roles in lipid storage and metabolism.

Rab proteins are evolutionarily conserved in many organisms. *Drosophila* has 31 Rabs, 23 of which have mouse and human orthologs [17]. Similar to mammals, lipids in *Drosophila* larvae are mainly stored in adipose tissue fat bodies. *Drosophila* lipid droplets are coated with the PAT domain proteins, PLIN1 and PLIN2. *plin2* mutants are lean, showing lower levels of TAG and small lipid droplets, while *plin1* mutants are adult-onset obese [18], [19]. *Drosophila* has been used extensively as a model organism in lipid metabolism studies [20], [21], [22], [23], [24], [25]. For example, a whole genome RNAi screen of S2 cells showed that about 1.5% of all the genes tested function in lipid droplet formation and utilization [26]. Another RNAi screen in adult flies identified about 500 obesity genes. The Hedgehog signaling pathway was shown to have a fat body-specific role in *Drosophila* and to function as a switch between brown and white adipose tissues in mammals, suggesting that fat storage mechanisms are conserved between *Drosophila* and mammals [27].

In this study, we have systemically investigated the potential roles of Rab proteins in the regulation of lipid storage in *Drosophila*. As a small GTPase, Rab protein can switch between its GDP-binding inactive form and GTP-binding active form. Guanine nucleotide exchange factor (GEF) switches GTPase from its inactive to its active form, while GTPase activating protein (GAP) inactivates GTPase. With the help of structural and functional analysis, specific amino acid changes can be made that keep Rab GTPase in its GDP-binding form (dominant negative/DN) or GTP-binding form (constitutive active/CA). Expression of the DN or CA form can therefore mimic loss-of-function or gain-of-function effects. To examine the effect on lipid droplets, we manipulated the activity of Rabs in the fat body using the *Gal4-UAS* system and a transgene collection of DN and CA forms of all 31 *Drosophila Rabs* [17]. Lipid droplet size changes were found in many *DN-* or *CA-Rab*-expressing larvae, suggesting that these Rabs may regulate the dynamics of lipid droplets. In particular, we analyzed the molecular function of Rab32 and Rab32 GEF/Claret in lipid storage in detail. We show that Rab32 may affect lipid storage through its effects on autophagy.

## Results

### Systematic identification of Rabs that affect the size of lipid droplets

**Figure 1:**
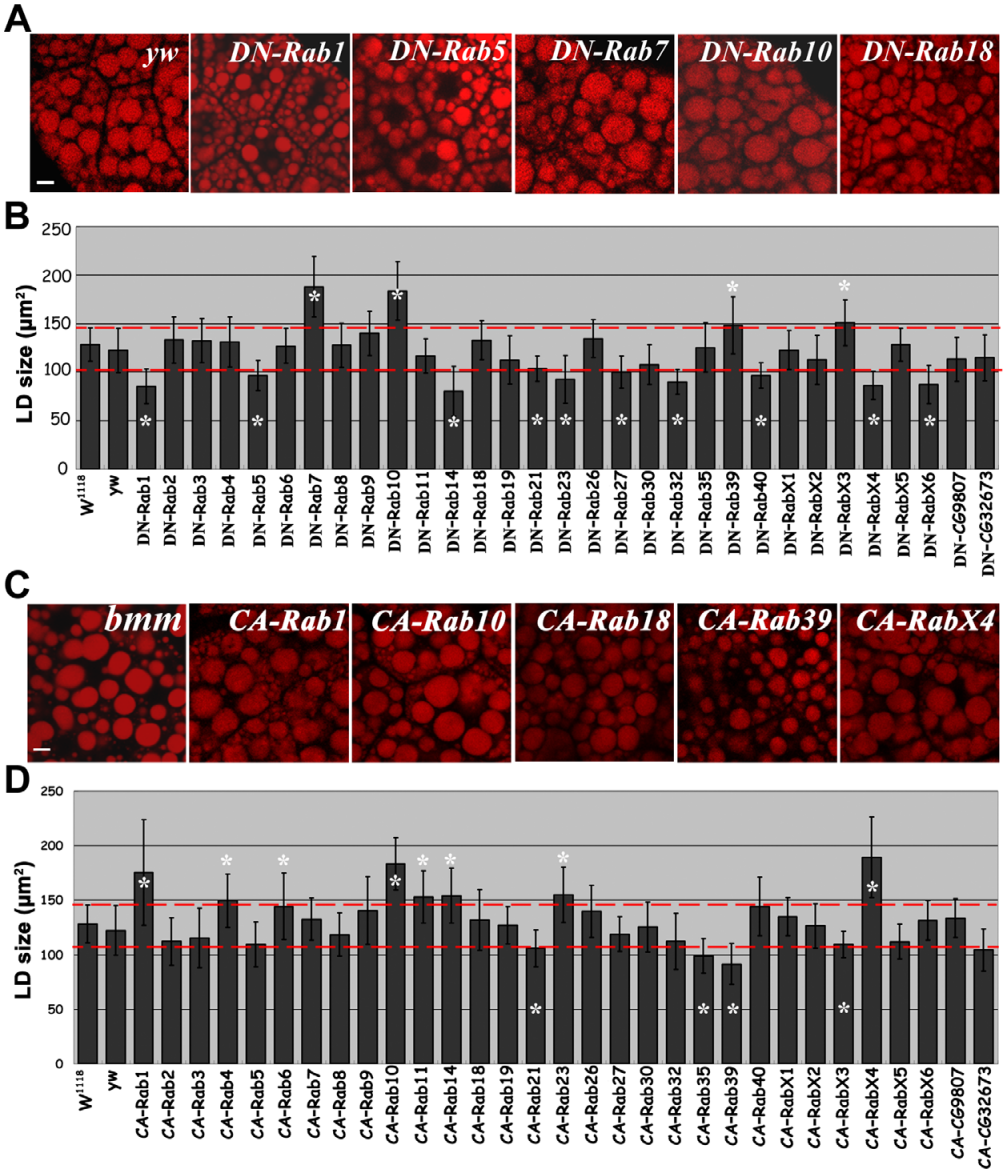
Genetic screen for Rabs that affect the size of lipid droplets. (A) Nile red staining of lipid droplets in wandering stage third instar larval fat body cells. Dominant-negative (DN) Rab expression reduces (DN-Rab1 and DN-Rab5) or increases (DN-Rab7 and DN-Rab10) lipid droplet size. Expressing DN-Rab18 does not affect the size of lipid droplets. Scale bar: 10 µm. (B) Quantification of the effects of all DN-Rabs. The error bars represent the standard deviation. Red dashed lines indicate the average variation in lipid droplet size in controls. *: P<0.01. (C) Nile red staining of lipid droplets in wandering stage third instar larval fat body cells. Constitutive-active (CA) Rab expression reduces (CA-Rab39) or increases (CA-Rab1, CA-Rab10, and CA-RabX4) lipid droplet size. Expressing CA-Rab18 does not affect the size of lipid droplets. *bmm* mutant was included as a comparison. Scale bar: 10 µm. (D) Quantification of the effects of all CA-Rabs. The error bars represent the standard deviation. Red dashed lines indicate the average variation in lipid droplet size in controls. *: P<0.01.

To systematically investigate the potential functions of Rabs in regulating lipid storage, we performed a functional screen by manipulating Rab activity in a tissue-specific manner using the *UAS-Gal4* system [28]. The fat body-specific *ppl-Gal4* was used to drive the expression of individual *UAS-DN-* or *CA-Rabs* [29]. We then used Nile red dye to stain the lipid droplets in the fat body of wandering stage third instar larvae. We found that expression of DN-Rabs 1, 5, 14, 21, 23, 27, 32, 40, X4, and X6 resulted in small lipid droplets, while expression of DN-Rabs 7, 10, 39, and X3 led to large lipid droplets (Fig. 1A and 1B). When CA-Rabs were expressed in the fat body, CA-Rabs 21, 35, 39, and X3 expression reduced the size of lipid droplets and CA-Rabs 1, 4, 6, 10, 11, 14, 23, and X4 expression increased lipid droplet size (Fig. 1C and 1D). Thus, the DN- and CA-forms of Rabs 1, 14, 23, 39, X3, and X4, had opposite effects on the size of lipid droplets. In addition, Rabs 4, 5, 6, 7, 11, 27, 32, 35, 40, and X6, only affected lipid droplet size when either the DN- or CA- form was expressed, but not when both were expressed (Table 1). Intriguingly, both DN- and CA- forms of Rab10 and Rab21 exhibited similar effects on lipid droplet size. Since RNAi stocks for most Rabs are available, we next investigated whether knockdown these Rabs lead to similar effects as DN-Rabs. Consistently, RNAi of Rab1, 5, 21, 40, X4, and X6 reduced lipid droplet size (Fig. S1). RNAi of Rab10, 23, 27 did not affect the size of lipid droplets, although the knockdown efficiency remained to be evaluated. In addition, except Rab23, most Rabs tested are strongly expressed in the larval fat body (Fig. S2). Taken together, these results support the involvement of many Rabs in lipid droplet size control.

**Table 1:** Comparison of Rabs identified in this study with previous proteomic studies [13], [14].

Proteomic studies (mammalian cells)	This study (*Drosophila*)
Rab1	Rab1
Rab2	No phenotype
Not Found	Rab4
Rab5	Rab5
Rab6	Rab6
Rab7	Rab7
Rab8	No phenotype
Rab10	Rab10
Rab11	Rab11
Rab14	Rab14
Rab18	No phenotype
Rab19	No phenotype
Rab21	Rab21
Not Found	Rab23
Rab24	Not available
Not Found	Rab27
Not Found	Rab32
Rab33	Not available
Rab34	Not available
Rab35	Rab35
Rab39	Rab39
Not Found	Rab40
Rab41	Not available
Not available*	RabX3
Not available	RabX4
Not available	RabX6

* Not available means the homolog can't be found.

In addition, we compared our genetic results with results from previous proteomic studies, in which a total of 18 Rabs were identified (Table 1) [14]. Four of these 18 Rabs have no orthologs in *Drosophila* (Table 1). In our screen, a set of 18 Rab proteins was found to affect the size of lipid droplets, of which Rabs X3, X4, and X6 have no counterparts in mammals. Importantly, we noticed that Rabs 1, 5, 6, 7, 10, 11, 14, 21, 35, and 39 were found in both previous proteomic studies and our functional screen (Table 1). The significant overlap between these results suggests that these Rabs may function directly on lipid droplets to regulate the dynamics of lipid droplets. Interestingly, although Rabs 2, 8, 18, and 19 were identified in proteomic studies, neither their DN- nor CA- forms altered lipid droplet size in our study (Table 1). These Rab proteins may play roles in aspects of lipid droplets other than their size. Alternatively, they may regulate the size of lipid droplets under unusual conditions, such as starvation. Rabs 4, 23, 27, 32, and 40 were found in our screen, though they had not been identified in proteomic reports (Table 1), implying that these Rabs may affect the size of lipid droplets indirectly.

### *Rab32/lightoid (ltd)* and *Rab32 GEF/claret (ca)* affect the size of lipid droplets

We next validated the screen results using mutant phenotypic analysis and then further investigated the functional mechanisms of Rabs in lipid droplet dynamics and lipid metabolism. We focused particularly on Rab32 for the following reasons. First, there are many more mutant alleles available for *Rab32* than for other *Rabs*. Second, Rab32 is highly expressed in the fat body, which is consistent with a potential role in regulating lipid metabolism (FlyAtlas: http://flyatlas.org, and Fig. S2). Lastly, *Rab32*, also known as *lightoid* (*ltd*), acts in a well-known eye pigment granule biosynthesis pathway and many components of this pathway have previously been identified [30], [31].

**Figure 2:**
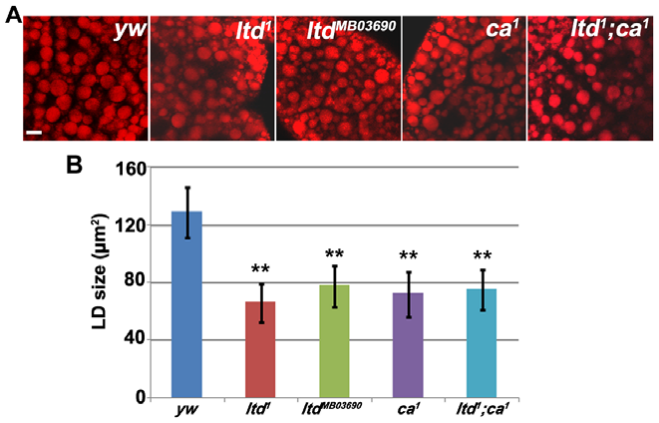
Lipid droplet size is reduced in *Rab32/ltd* and *Rab32 GEF/ca* mutants. (A) Nile red staining of lipid droplets in wandering stage third instar larval fat body cells. *Rab32/ltd* and *Rab32 GEF/ca* mutants have smaller lipid droplets compared to the control. Scale bar: 10 µm. (B) Quantification of the effects of *Rab32/ltd* and *Rab32 GEF/ca* single mutants and *ltd;ca* double mutants. The error bars represent the standard deviation. **: P<0.001.

We found that the *ltd* loss-of-function mutants, *ltd^1^* and *ltd^MB03690^*, had smaller lipid droplets than controls (Fig. 2A and 2B). This is consistent with DN-Rab32 expression results (Fig. 1A). Moreover, fat body specific expression of *Rab32* can fully rescue the small lipid droplet phenotype of *ltd^1^* (Fig. S2). Claret has been reported as a GEF for Ltd and is essential for the activation of Ltd. *claret* (*ca*) mutants show a similar defect to *ltd* mutants in the eye pigment granule biogenesis process [30]. As was the case for *ltd* mutants, *ca* mutants also had a small lipid droplet phenotype (Fig. 2A and 2B). In addition, *ltd;ca* double mutants exhibited the same small lipid droplet phenotype as both single mutants, consistent with a previous finding that they function together in the same pathway (Fig. 2A and 2B). Therefore, we conclude that both *Rab32/ltd* and *Rab32 GEF*/*ca* are required for maintaining normal lipid droplet size. These results also validated the results of our functional screen.

### *ltd*, *ca*, and *rb* are required for normal lipid storage

**Figure 3:**
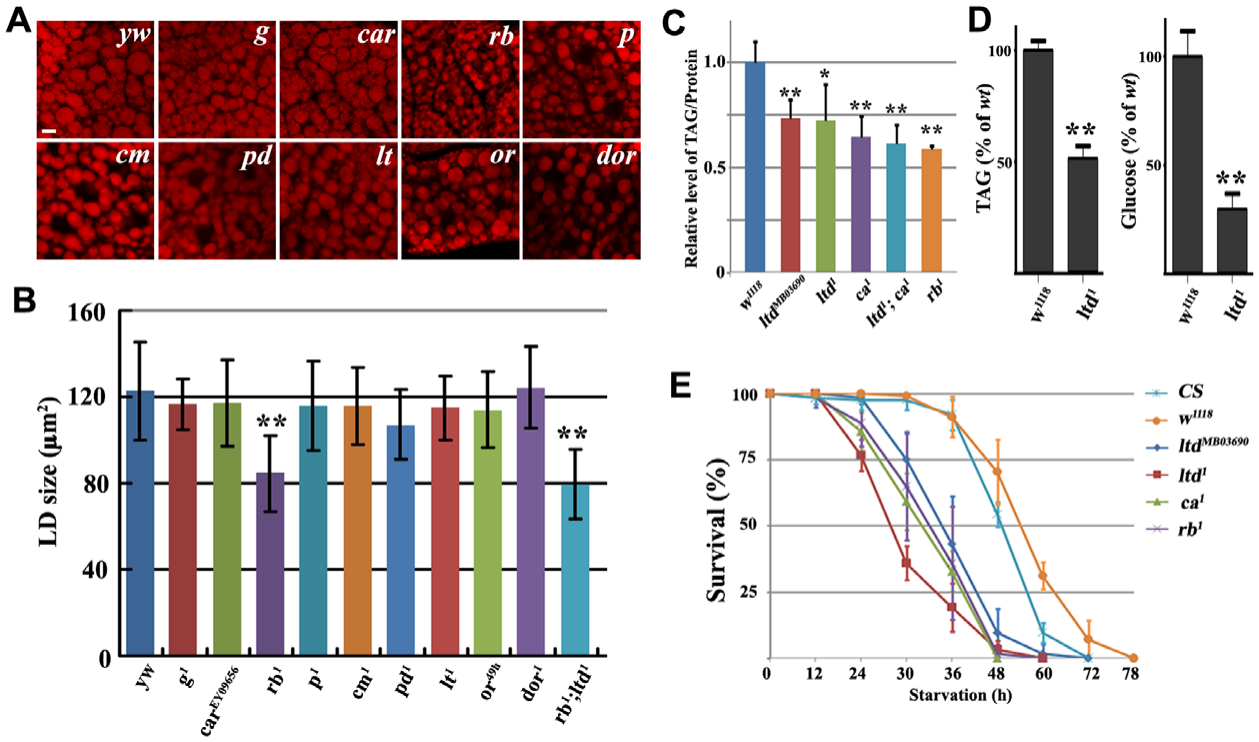
*ltd*, *ca* and *rb* affect lipid storage. (A) Lipid droplets in different eye pigment granule biogenesis mutants. Nile red staining of lipid droplets in wandering stage third instar larval fat body cells. *rb* mutants have small lipid droplets. Scale bar: 10 µm. (B) Quantification of lipid droplet size in different eye pigment granule biogenesis mutants and *rb;ltd* double mutants. The error bars represent the standard deviation. **: P<0.001. (C) The level of TAG is reduced in *ltd*, *ca* and *rb* mutant larvae. The error bars represent the standard deviation. *: P<0.01; **: P<0.001. (D) The level of TAG and glucose are reduced in *ltd^1^* mutant adults. **: P<0.001. (E) Survival curve of adult flies under starvation. *ltd*, *ca* and *rb* mutants are sensitive to starvation.

Eye pigment granules in *Drosophila* are specialized types of lysosome-related organelles. The genetic pathway of eye pigment granule biogenesis has been well studied in *Drosophila*. In addition to *ltd* and *ca*, at least nine more genes, including *garnet* (*g*), *carnation* (*car*), *ruby* (*rb*), *carmine* (*cm*), *purploid* (*pd*), *deep orange* (*dor*), *orange* (*or*), *light* (*lt*) and *pink* (*p*), are also required in this process [31]. We examined larval fat bodies in mutants of these nine genes and found that only *rb* mutants have small lipid droplets (Fig. 3A and 3B), indicating that the regulation of lipid droplet size may share some components with, but may not be identical to, the eye pigment granule biogenesis pathway. Furthermore, *rb;ltd* double mutants have the same small lipid droplet phenotype as *rb* single mutant, suggesting that *ltd* and *rb* act similarly in regulating lipid storage (Fig. 3B).

Changes in lipid droplet size in *ltd*, *ca*, and *rb* mutants may reflect changes in lipid levels. We measured larval TAG levels and found that all three mutants had lower TAG levels compared to the wild type (Fig. 3C). For example, the TAG level in the *ltd^1^* and *ltd^MB0369^* mutant larvae was only ∼70% that of the wild type. Moreover, *ltd;ca* double mutants had a similar lipid content to that of the single mutants. In addition, the levels of TAG and glucose are significantly reduced in *ltd^1^* mutant adults compared to the wild type (Fig. 3D). These results suggest that *ltd*, *ca*, and *rb* are necessary for lipid storage.

Another piece of evidence suggesting that *ltd*, *ca*, and *rb* function in lipid storage comes from starvation tests. Under starved conditions, animals can mobilize stored lipids from the lipid droplet by lipolysis for energy consumption. Animals with elevated TAG levels may be resistant to starvation, while animals with decreased TAG levels may be sensitive to starvation. We found that *ltd*, *ca*, and *rb* mutants were more sensitive to starvation than controls. For instance, nearly all the mutant animals were dead after a 48-hour period of starvation, while around 50% of the control animals were still alive (Fig. 3E). These results further confirm that lipid storage is impaired in these mutants and that lipid levels are decreased.

### *ltd* and *ca* genetically interact with *plin2* and *bmm*

**Figure 4:**
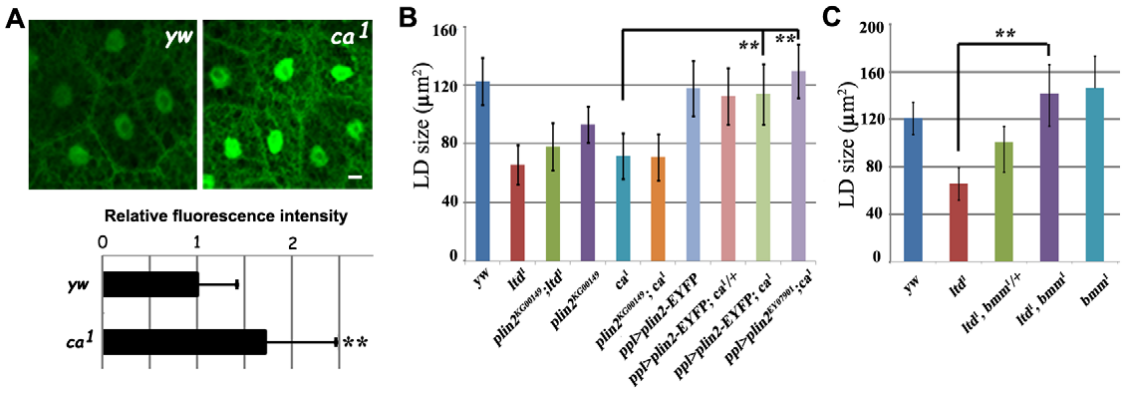
*ltd* and *ca* genetically interact with the two lipolysis-related genes *bmm* and *plin2*. (A) Image and fluorescence intensity quantification of lipogenic reporter Gal4-SREBP>UAS-GFP in *ca* mutants and control. Scale bar: 20 µm. **: P<0.001. (B) Quantification of lipid droplet size in different genetic backgrounds. *ltd*;*plin2* or *ca*;*plin2* double mutants did not show an enhanced phenotype compared to single mutants. Overexpressing *plin2* suppresses *ca*. The error bars represent the standard deviation. **: P<0.001. (C) Quantification of lipid droplet size in different genetic backgrounds. *bmm* mutants suppress *ltd* mutants in a dosage-dependent manner. The error bars represent the standard deviation. **: P<0.001.

What is the mechanism by which Rab32 affects lipid storage? Both increased lipolysis and reduced lipogenesis may lead to the reduced lipid storage phenotype in *Rab32*/*ltd* and its *GEF ca* mutants. Using a Gal4-SREBP>UAS-GFP fluorescent reporter system which has been used as an indicator of lipogenesis in *Drosophila* [32], we found that lipogenesis is not reduced in *ca* mutants (Fig. 4A).

The lean phenotype in *ltd*, *ca*, or *rb* mutants resembles that of *plin2* mutants. PLIN2 is a PAT domain protein localized on the surface of lipid droplets and is involved in lipolysis. *plin2* mutants show a reduction in TAG level and small lipid droplets, likely due to increased lipolysis (Fig. 4B) [19], [33]. We tested the genetic interaction between *plin2* and *ltd* or *ca*. Lipid droplets in *plin2;ca* double mutants were the same size as those of the *ca* single mutant. *plin2;ltd* double mutants yielded similar results, suggesting that *plin2* and *ltd* or *ca* may affect the same process (Fig. 4B). Moreover, fat body-specific expression of *plin2* using either the *UAS-plin2-EYFP* transgene line or the *plin2* EP line (*EY07901*) significantly rescued the *ca* mutant phenotype (Fig. 4B). These results indicate that *Rab32 GEF/ca* genetically interacts with *plin2* and *Rab32* and may ultimately affect lipolysis.

*brummer* (*bmm*), the *Drosophila* homolog of the mammalian adipocyte triglyceride lipase (ATGL) gene, is an important fat storage regulator in *Drosophila*. *bmm* mutants have reduced lipolysis, accumulations of TAG, and enlarged lipid droplets (Fig. 4C) [33]. Since *Rab32*/*ltd* likely acts on lipolysis, we tested its genetic interaction with *bmm*. We generated *ltd;bmm* double mutants and found that mutation of *bmm* could suppress the *ltd* mutant phenotype in a dose-dependent manner. Removal of one copy of *bmm* in *ltd* mutants significantly suppressed the small lipid droplet phenotype. Lipid droplet size in *ltd;bmm* double mutants was similar to that in *bmm* mutants (Fig. 4C). This dose-dependent suppression of *ltd* by *bmm* further supports that *Rab32*/*ltd* may modulate lipolysis.

### Rab32 is localized in the lysosome and/or autophagosome

**Figure 5:**
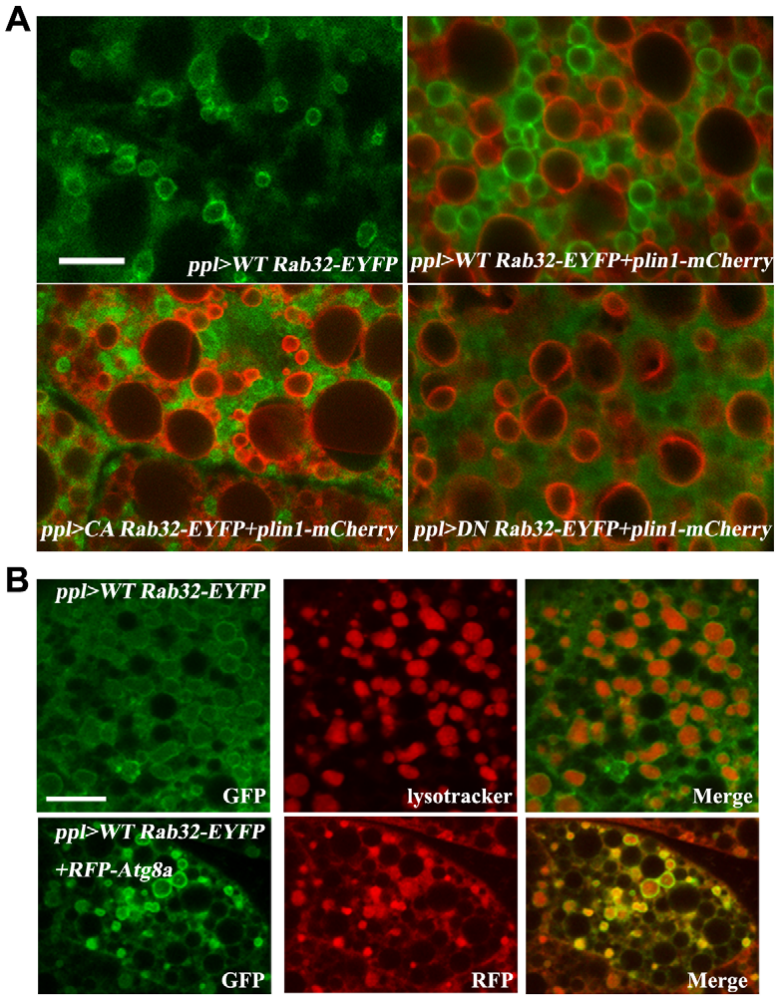
Rab32 is localized to the lysosome and autophagosome. (A) The localization of WT, CA- and DN-Rab32-EYFP in wandering stage third instar larval fat body cells. WT and CA-Rab32-EYFP are localized to ring-like structures and do not colocalize with the lipid droplet marker PLIN1-mCherry. DN-Rab32-EYFP is located in the cytosol. Scale bar: 20 µm. (B) WT Rab32-EYFP appears as rings surrounding lysotracker-labeled lysosomes and RFP-Atg-8-marked autophagosomes. Scale bar: 20 µm.

We next examined the subcellular localization of Rab32 in the fat body. Rab32 exhibited a ring-like localization pattern when we used *ppl-Gal4* to drive *UAS-Rab32-EYFP* expression (Fig. 5A). In addition, *ppl-Gal4*-driven *UAS-CA-Rab32-EYFP* showed a similar localization pattern, while the ring-like localization pattern was lost in *ppl-Gal4*-driven *UAS-DN-Rab32-EYFP*. The DN-Rab32-EYFP signal was diffuse throughout the cytosol. These results indicate that Rab32 is localized on the surface of some vesicles/organelles and that its location may be critical for its normal function. Are these vesicles/organelles lipid droplets? We used PLIN1-mCherry to label lipid droplets [25], and found that Rab32-EYFP did not co-localize with PLIN1-mCherry at all (Fig. 5A), indicating that it is not located in lipid droplets. These findings are consistent with the fact that Rab32 was not found in previous proteomic studies and suggest that Rab32 affects lipid storage in organelles other than lipid droplets.

Since Rab32 is known to function in the biogenesis of the eye pigment granule [30], which is a lysosome-related organelle, we wondered whether Rab32 is localized to the lysosomes or lysosome-related organelles. We found that Rab32-EYFP co-localizes perfectly with lysotracker, suggesting that Rab32 is localized in the lysosome or lysosome-related organelles in fat body cells (Fig. 5B).

During insect metamorphosis, the fat body, salivary gland, and midgut undergo programmed autophagy. Programmed autophagy is induced by ecdysone at the L3 stage. At the early L3 stage, puncta of Atg5 and Atg8 (autophagy markers) are found in the fat body, indicating the formation of the autophagosome. At the late L3 stage, autophagosomes fuse with lysosomes and can be labeled by both lysosomal markers and autophagosome markers [34]. To determine whether the ring-like Rab32-positive vesicles observed here were autophagosomes, we co-expressed RFP-Atg8a with Rab32-EYFP in the fat body. We found that the RFP-Atg8a-labeled autophagosomes were coated by Rab32-EYFP (Fig. 5B). These results indicate that Rab32 is localized in autophagosomes and suggest that Rab32 may have a potential regulatory function in autophagy.

### Autophagy is impaired in *ltd* and *ca* mutants and defective autophagy leads to small lipid droplets

**Figure 6:**
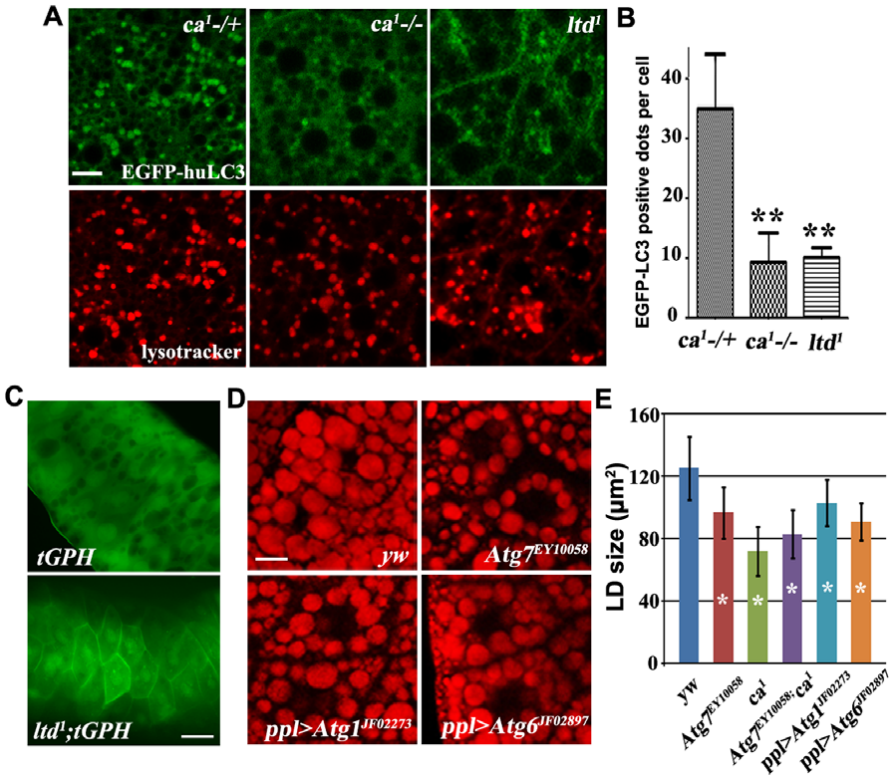
Autophagy is impaired in *ca* mutants and defective autophagy leads to small lipid droplets. (A) Autophagy marker EGFP-huLC3-positive puncta are greatly reduced in *ltd^1^* and *ca^1^* homozygous mutants compared to the *ca^1^* heterozygous control. Scale bar: 20 µm. (B) Quantification of EGFP-huLC3-marked autophagosomes in *ca^1^* heterozygous and *ltd^1^* and *ca^1^* homozygous mutants. The error bars represent the standard deviation. **: P<0.001. (C) tGPH reporter in control and *ltd^1^* mutant salivary glands at 13.5 hr after pupae formation. tGPH cortical signal persists in *ltd^1^* mutants. Scale bar: 50 µm. (D) Nile red staining of lipid droplets in wandering stage third instar larval fat body cells. Autophagy mutants or *Atg1* and *Atg6* RNAi animals have small lipid droplets. Scale bar: 20 µm. (E) Quantification of lipid droplet size in different genetic backgrounds. The error bars represent the standard deviation. *: P<0.001.

To address whether Rab32 activity is required for autophagy in larval fat body, we labeled autophagosomes with GFP-huLC3. huLC3 is the human homolog of Atg8 and has been widely used as an autophagosome marker in autophagy activity assays [34]. GFP-huLC3-positive structures were also labeled by lysotracker (Fig. 6A and 6B). We observed many GFP-huLC3-positive and lysotracker-positive autophagosomes in *Rab32 GEF ca* heterozygous animals, indicating that autophagy was normal. However, in *ltd* or *ca* homozygous mutant animals, the number of GFP-huLC3-positive autophagosomes was dramatically reduced, indicating that the autophagy process was impaired in these mutants (Fig. 6A and 6B). These data suggest that Rab32 activity is required for the autophagy process of fat body at the late L3 stage. We further examined whether Rab32 regulates autophagy in other tissues. Salivary gland is an ideal in vivo system for studies of autophagic cell death [35]. Normally, the cortical tGPH (tubulin-GFP-Pleckstrin-Homology) signal is lost at 13.5 hr after pupae formation, the time of salivary gland cell death (Fig. 6C). However, in *ltd* mutants, the cortical signal persists in the salivary gland, indicating autophagic cell death defects (Fig. 6C). Together, these results suggest that autophagy is impaired in *ltd* and *ca* mutants.

Several previous studies provide supporting evidence for a connection between autophagy and lipid storage as well as lipolysis. For example, knock-out of either *Atg5* or *Atg7* leads to reduced lipid accumulation and impaired adipocyte differentiation in mice [36], [37]. We wondered whether down-regulation of autophagy in *Drosophila* could also lead to a similar effect. We analyzed *Atg7* loss-of-function mutants and found that the size of lipid droplets was smaller in the mutants than in controls (Fig. 6D and 6E). Additionally, *Atg7;ca* double mutants did not exhibit an enhanced phenotype, indicating that *Atg7* and *ca* likely act in the same genetic pathway (Fig. 6D and 6E). To further confirm this result, we used *ppl-Gal4* to drive *UAS-Atg1* or *Atg6* RNAi to specifically knock-down these genes in fat body cells. Knock-down of *Atg1* or *Atg6* led to small lipid droplets, suggesting an essential role of autophagy in lipid storage in fat body cells (Fig. 6D and 6E). Therefore, we concluded that Rab32 may regulate lipid storage by affecting autophagy.

## Discussion

Lipid droplets are the main storage sites of neutral lipids in all cells, however, the dynamics of lipid droplets are poorly characterized. Here we systematically investigated the functions of all of the 31 *Drosophila* Rabs in the dynamics of lipid droplets and lipid storage by expressing their DN- and CA- forms. Eighteen Rabs were identified, 10 of which, including Rab1, had been found in previous proteomic studies. Rab1 is important for ER to Golgi transport by tethering the COPII-coated vesicles to Golgi through it effector p115 [38]. Interestingly, it was reported that COPI and COPII involved pathway delivers ATGL to lipid droplets to mediate lipolysis [39]. Five Rabs are not present in previous proteomic lists. These Rabs may not act on lipid droplets directly and instead may act on other organelles to influence lipid storage. Rab32 is an example of one of these Rabs.

*Rab32*/*ltd* and its *GEF ca* have well known functions in the biogenesis of a specific type of lysosome-related structure, called the eye pigment granule [30]. Many mutants have been found which have defective eye pigments. Proteins encoded by these genes include enzymes required for eye pigment biogenesis, ABC transporters responsible for the trafficking of pigment precursors, and the so-called “granule group” [31]. Four granule group genes, *g*, *car*, *or*, and *rb*, encode homologs of different AP-3 subunits which are believed to be involved in protein trafficking into lysosomes. Among them, only *rb* is required for lipid storage (Fig. 3A), suggesting that AP-3 subunits may have different roles in the regulation of lipid metabolism.

The regulation of lipid storage involves both the biosynthesis and the usage of lipids. Lipids are mainly stored in the lipid droplet, a monolayer-membrane-bound organelle, which is different structurally from lysosomes and lysosome-related organelles. Our studies of Rab32 reveal that the lysosomal pathway and the regulation of lipid storage may converge at points such as lipolysis. One explanation is that Rab32 may function in these different processes in a similar way. Alternatively, *Rab32* could affect the lysosome and lysosome-related processes, subsequently influencing lipid storage.

Our results support the second possibility. First, we found that Rab32 is localized in autophagosomes, but not lipid droplets. Its location appears to be important for Rab32 function, since the DN form of Rab32 is mainly present in the cytosol. Second, it is known that autophagy affects lipid storage [40], [41]. The autophagosome is a special lysosome-related organelle. Lipid storage is reduced in the adipocytes of mice autophagy mutants [36], [37]. Third, levels of the autophagy activity marker GFP-huLC3 are reduced in *Rab32* and *ca* mutants, suggesting that *Rab32* and *ca* mutants have impaired autophagy. Therefore, Rab32 may execute its functions in lipid storage by affecting autophagy. Lastly, in mice, Rab32 and Rab38, which is very closely related to Rab32, have different expression patterns and function redundantly in the biogenesis of the melanosome, which is also a lysosome-related organelle [42]. In cultured cells, human Rab32 affects the formation of autophagic vacuoles [43]. These results suggest that the functions of Rab32 are likely evolutionarily conserved.

What is the relationship between autophagy and lipid storage? Previously, several studies reported that autophagy can regulate lipid metabolism. For example, inhibition of autophagy in cultured hepatocytes by *Atg5* RNAi or 3-methyladenine, an autophagy inhibitor, leads to increased TAG storage in lipid droplets [40]. In addition, hepatocyte-specific knockout *ATG7* results in elevated hepatic lipids [41]. Interestingly, in contrast, knockdown of *ATG5* or *ATG7* in the pre-adipocyte cell line 3T3-L1 leads to decreased TAG accumulation, affecting adipocyte differentiation [36], [37]. In vivo, adipocyte-specific knockout *ATG7* mice are lean and have greatly reduced white adipocyte mass, but increased brown adipocyte mass. Mutant white adipocytes exhibit features resembling brown adipocytes, such as an increased rate of fatty acid β-oxidation, suggesting that autophagy may affect adipocyte differentiation [37]. Therefore, autophagy may affect lipid metabolism in a tissue-specific manner.

Our results also support the involvement of autophagy in lipid metabolism. During the wandering third instar larvae to pupae transition, animals do not feed and are in a state resembling starvation or nutrient-deprivation. Programmed autophagy of fat bodies and other tissues is important for providing energy and other nutrients for development. Mutations in or tissue-specific knockdown of autophagy components lead to reduced lipid storage (Fig. 6C and 6D). These results suggest that in autophagy mutants, more lipids may be mobilized from lipid droplets to compensate for the shortage of energy. It is conceivable that by affecting autophagy, Rab32 likely regulates lipid storage through lipolysis. The genetic interactions between *Rab32/ltd* and lipolysis-related genes, *bmm* and *plin2*, further support this hypothesis. This study has highlighted the potential functions of Rabs in regulating lipid metabolism. Further studies will elucidate the intermingled relationship between autophagy and lipid metabolism during development.

## Materials and Methods

### Fly strains

*Drosophila* stocks were maintained in standard corn meal food, unless specified. *Canton-S (CS)*, *w^1118^* or *yw* were treated as controls. *bmm^1^* mutants were kindly provided by Dr. Ronald P. Kühnlein [33]. The P*{GAL4-dSREBPg}* transgene was kindly provided by Dr. Robert B. Rawson. The *UAS-RFP-Atg8a* transgene was kindly provided by Dr. Ernst Hafen [44]. *UAS-plin1-mCherry* and *UAS-plin2-EYFP* transgene lines were generated using a standard protocol. All other strains were obtained from the Bloomington Stock Center.

### Fat body dissection and imaging

Wandering stage third instar larvae were dissected in 1xPBS, fixed with 4% paraformaldehyde for 75 min, washed twice with 1xPBS, and then stained with Nile red (0.5 µg/ml) or Bodipy 493/503 (1 µg/ml) for 60 min. After washing twice with 1xPBS, the samples were observed under a confocal microscope. The sizes of lipid droplets were measured using NIS-Elements BR 3.0 software (Nikon). The five largest lipid droplets in every fat body cell were measured and their average size was counted as the lipid droplet size for each cell. A total of 30 cells from at least 3 images were measured for each genotype. The lipogenic reporter Gal-4-SREBP>UAS-GFP fluorescence images were taken using a confocal microscope. Mean fluorescence intensity from 50 fat body cells were compared between mutants and control. Lysotracker (Molecular Probes) staining was performed according to the manufacturer's instructions. Briefly, wandering stage third instar larvae were dissected in 1xPBS and incubated with 10 µm Lysotracker red DND-99 before observing immediately under a confocal microscope. Salivary gland cortical tGPH signal at 13.5 hr after pupae formation was captured as previously described [35]. For quantification purpose, images were taken using equivalent exposure conditions for controls and mutants. A two-tailed Student's *t*-test was used to determine the significance of differences.

### Starvation tests

3-day old adult males were used in starvation tests. Twenty-five flies were placed in a vial and fed with only water for the duration of the starvation period. The survival ratio was recorded every 6 or 12 hours. Five replicate starvation tests were performed for each genotype.

### TAG and glucose level measurements

TAG levels were measured as previously described [25]. Briefly, 10 wandering stage third instar larvae or 5 3-day old adult males per genotype were homogenized in 100 µl PBT (PBS, 0.1% Tween 20) and immediately incubated at 75°C for 15 min. Samples were centrifuged for 3 min. Supernatants (20 µl) were treated with either 20 µl PBS or Triglyceride Reagent (Sigma) for 30 min at 37°C. 30 µl of each sample was then transferred to a 96-well plate and incubated with 100 µl of Free Glycerol Reagent (Sigma) for 5 min at 37°C. Samples were assayed at 540 nm using a spectrophotometer. TAG levels were determined by subtracting the amount of free glycerol in the PBS-treated sample from the total glycerol in the sample treated with Triglyceride Reagents. At least 3 repeat measurements were made for each genotype. To compare the relative TAG levels in different genotypes, TAG levels were normalized with the protein level from the same sample. Protein concentrations were determined using Bradford Reagent (Sigma). Glucose levels were measured similarly using Sigma reagent [23].

## Supporting Information

Figure S1 **RNAi validation of Rabs that affect the size of lipid droplets.** (A) Bodipy staining of lipid droplets in wandering stage third instar larval fat body cells. Scale bar: 10 µm. (B) Quantification of the effects of Rab RNAi. ***: P<0.001.
(TIF)

Figure S2 **Rab32 is expressed and functions in the fat body.** (A) The fat body expression of *ca*, *rb*, and several *Rabs* analyzed by RT-PCR. (B) Bodipy staining of lipid droplets in wandering stage third instar larval fat body cells. The fat body specific expression of *Rab32* by *ppl-Gal4* driver can rescue the *ltd^1^* lipid droplet phenotype. Scale bar: 10 µm. (C) Quantification of the rescuing effect. **: P<0.001.
(TIF)
