# Supplementary figures and images for: Rab32 Is Important for Autophagy and Lipid Storage in Drosophila
Source: PLoS One. 2012 Feb 14;7(2):e32086. doi: 10.1371/journal.pone.0032086 (PMC3279429; doi:10.1371/journal.pone.0032086)

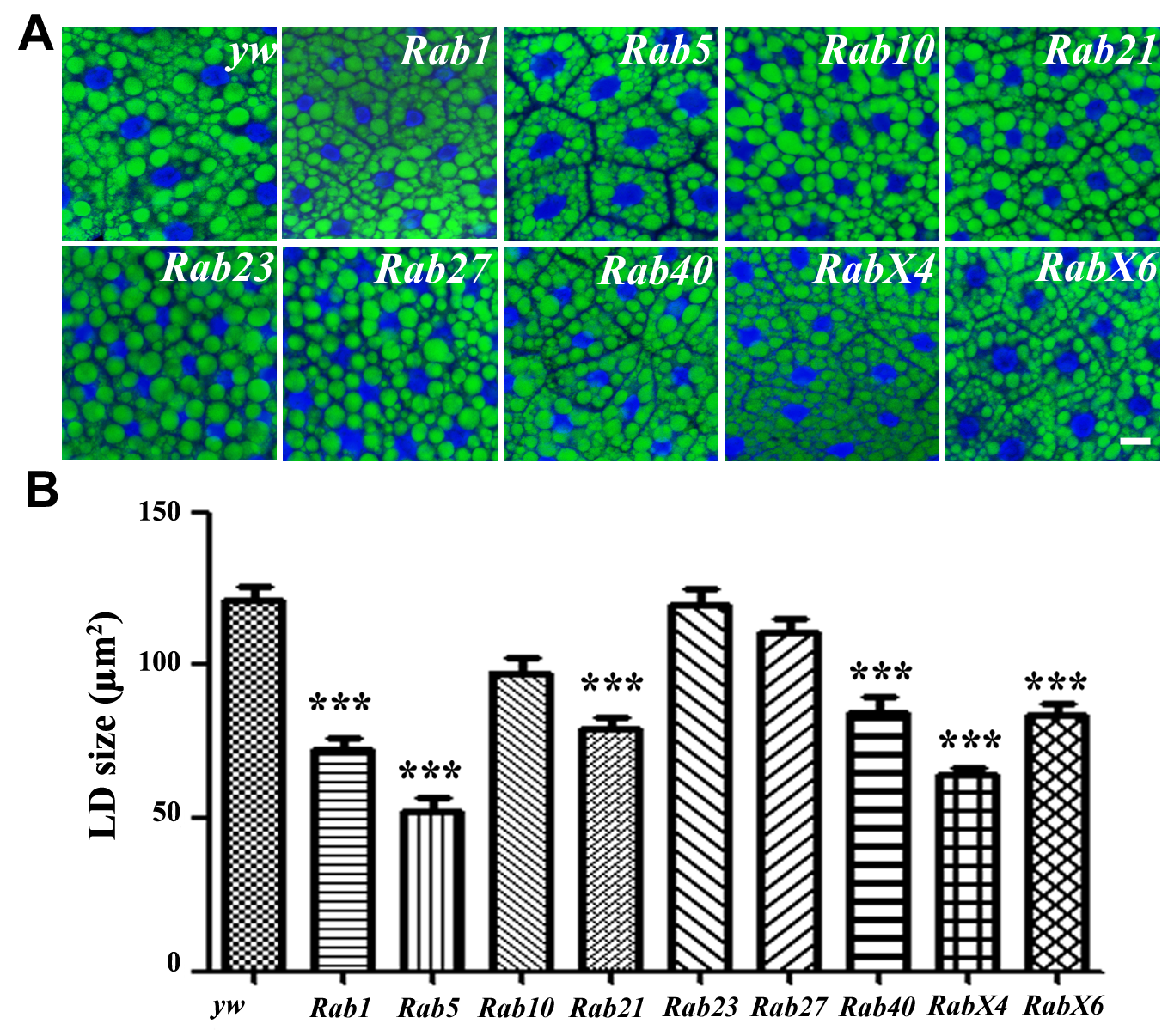

Supplement: Figure S1 — RNAi validation of Rabs that affect the size of lipid droplets. (A) Bodipy staining of lipid droplets in wandering stage third instar larval fat body cells. Scale bar: 10 µm. (B) Quantification of the effects of Rab RNAi. ***: P<0.001. (TIF) [file pone.0032086.s001.tif]

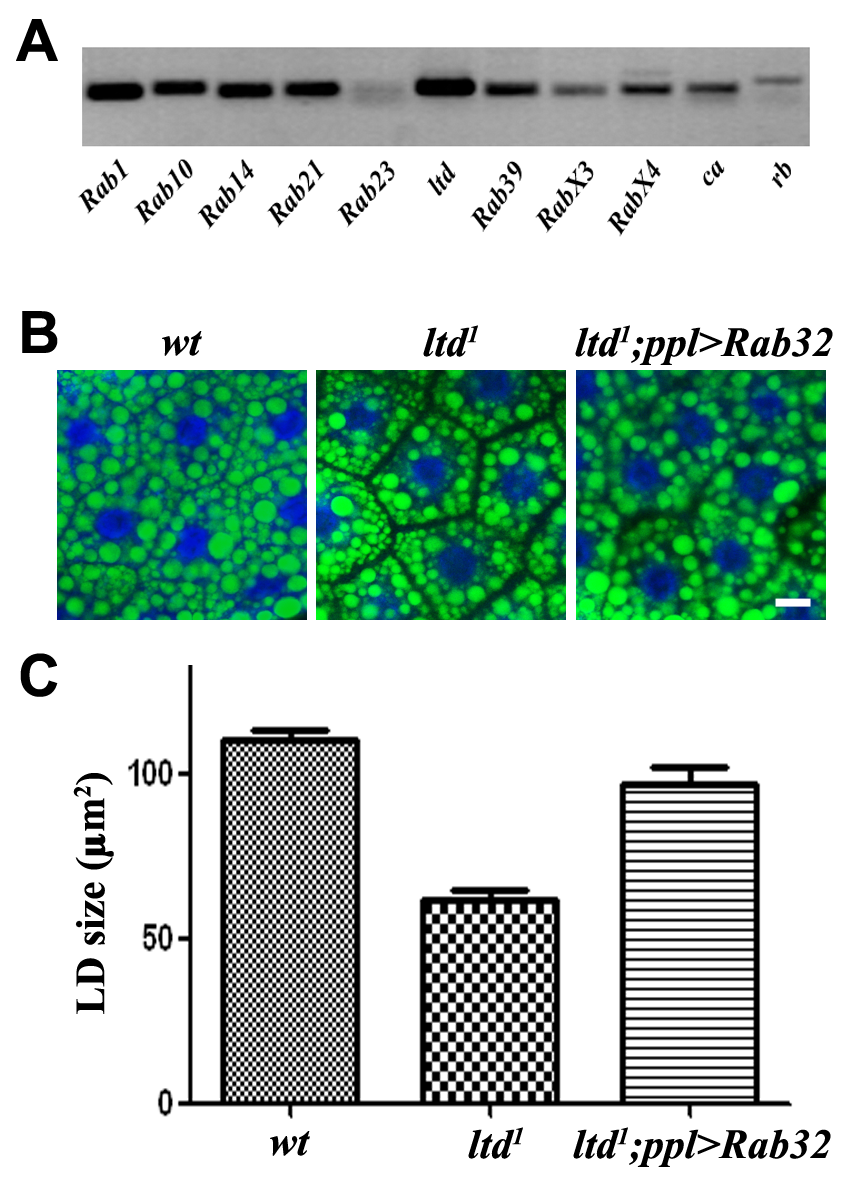

Supplement: Figure S2 — Rab32 is expressed and functions in the fat body. (A) The fat body expression of ca, rb, and several Rabs analyzed by RT-PCR. (B) Bodipy staining of lipid droplets in wandering stage third instar larval fat body cells. The fat body specific expression of Rab32 by ppl-Gal4 driver can rescue the ltd1 lipid droplet phenotype. Scale bar: 10 µm. (C) Quantification of the rescuing effect. **: P<0.001. (TIF) [file pone.0032086.s002.tif]
